# Early Fluid Resuscitation by Lactated Ringer’s Solution Alleviate the Cardiac Apoptosis in Rats with Trauma-Hemorrhagic Shock

**DOI:** 10.1371/journal.pone.0165406

**Published:** 2016-10-25

**Authors:** Kuan-Ho Lin, Chien-Liang Liu, Wei-Wen Kuo, Catherine Reena Paul, Wei-Kung Chen, Su-Ying Wen, Cecilia Hsuan Day, Hsi-Chin Wu, Vijaya Padma Viswanadha, Chih-Yang Huang

**Affiliations:** 1 College of Medicine, China Medical University, Taichung, 40402, Taiwan; 2 Department of Emergency Medicine, China Medical University Hospital, Taichung, Taiwan; 3 School of Chinese Medicine, College of Chinese Medicine, China Medical University and Hospital, Taichung, Taiwan; 4 Department of Biological Science and Technology, China Medical University, Taichung, Taiwan; 5 Graduate Institute of Basic Medical Science, China Medical University, Taichung, Taiwan; 6 Department of Dermatology, Taipei City Hospital, Renai Branch, Taipei, Taiwan; 7 Center for General Education, Mackay Junior College of Medicine, Nursing, and Management, Taipei, Taiwan; 8 Department of Nursing, MeiHo University, Pingtung, Taiwan; 9 Department of Biotechnology, Bharathiar University, Coimbatore-641 046, India; 10 Graduate Institute of Chinese Medical Science, China Medical University, Taichung, Taiwan; 11 Department of Health and Nutrition Biotechnology, Asia University, Taichung, Taiwan; Hospital Sirio-Libanes, BRAZIL

## Abstract

Cardiac trauma has been recognized as a complication associated with blunt chest trauma involving coronary artery injury, myocardium contusion and myocardial rupture. Secondary cardiac injuries after trauma supposed to be a critical factor in trauma patients, but the mechanism is not fully explored. Overproduction of TNF-alpha had been reported in multiple trauma animals, this induces oxidative stress resulting in cardiac apoptosis. Apoptosis gradually increases after trauma and reaches to a maximum level in 12 h time. TNF-alpha increases the expression of NFkB, and induces the expression of caspase-3 and resulted in cell apoptosis. The effect can be attenuated by non-selective caspase inhibitor and IL10. Fas induced cardiac apoptosis and hypertrophy in ischemic heart disease. In this study, we demonstrated a trauma-hemorrhagic shock (THS) model in rats and resuscitated rats by lactated Ringer’s (L/R) solution after shock in different hours (0 hour, 4 hours, 8 hours). NFkB gradually increased after the first 8 hours of shock, and can be reduced by fluid resuscitation. NFkB is known as a downstream pathway of Fas related apoptosis, we found Fas ligand, caspase-8 levels elevate after shock, and can be reduced by resuscitation. In addition, resuscitation can activate insulin-like growth factor (IGF-1)/Akt pathway, at the same time. It can block mitochondrial damage by decrease the effect of tBid. In conclusion, THS can induce secondary cardiac injury. Fas showed to be an important element in caspase cascade induced myocardium apoptosis. By L/R fluid resuscitation, the suppression of caspase cascade and activation of IGF-I/Akt pathway showed antiapoptotic effects in traumatic heart of rats.

## Introduction

Trauma-hemorrhagic shock (THS) is one among the most common causes of traumatic death [1]. Based on pre-hospital trauma life support in many countries, on-scene fluid resuscitation has been emphasized as a procedure to improve the survival rate [2, 3]. The benefits of fluid resuscitation for THS, such as the restoration of tissue perfusion, prevention of tissue hypoxia, attenuation of cytokine effect, and reduction of tissue apoptosis has been reported in many clinical and basic studies [4].

Secondary cardiac injury after trauma is considered to be a critical factor in trauma patients. It is also related to poor survival rate and prognosis [5]. Large amounts of Lactated Ringer’s solution (pH 6.6; sodium 130 mEq/L; potassium 4 mEq/L; calcium 3 mEq/L; chloride 109 mEq/L; lactate 28 mEq/L) is more often administrated in patients with severe hemorrhage to maintain the intravascular and extracellular fluid volumes and electrolyte balance. Lactated Ringer's is considered as an increment or replacement to whole blood as it is readily available, inexpensive, and free from infection [6]. The mechanism of hemorrhagic shock induced myocardial damage is not understood thoroughly, but apoptosis is thought to play an important role. THS and resuscitation activates myocardial NFkB and TNFα. NF-κB, is a transcription factor that is normally present in an inactive complex form consisting of p50 and p65 subunits along with the inhibitory protein, IκB-α. When stimulated, IκB-α is phosphorylated by IκB kinases and undergoes a rapid proteasomal degradation causing subsequent detachment of NF-κB from its inhibitors [7]. NFkB in turn translocates into nucleus and promotes the transcription of TNFα which is a myocardial suppressant that weakens cardiac contractile function, induces cardiac myocyte apoptosis, causes cardiac hypertrophy [8]. TNFα is the most important proapoptotic molecule that causes posttraumatic cardiomyocyte apoptosis [9]. Fas/fas ligand (fasL) simulates apoptosis in various cells, like lymphocytes, hepatocyte, and cardiomyocytes. When Fas/fasL binds to fas on fas-sensitive damaged cells, the subsequent activation of caspase cascade, results in apoptosis [10]. This mechanism is well-established in congestive heart failure, and ischemic heart disease. It is recognized as a predictor of adverse prognosis [11].

Cardiomyocytes undergo apoptosis by various stimulation, such as hypoxia, reoxygenation, acidosis, stretch, TNF-a, and Fas ligand. Ischemic tissue reperfusion injury is associated with elevated ROS production and Ca^2+^ overload [12]. Apoptosis is a programmed cell death, which is induced by two types of mechanisms. The first one is the extrinsic pathway, which is fas or TNF-α dependent. When fasL binds to receptor, the formation of fas-associated death domain (FADD) begins and the FADD recruits and activates the pro-caspase-8 and caspase-3 and thereby triggers apoptosis. The second one is intrinsic pathway, mitochondria dependent in which the balance of proapoptic protein, such as Bad (bcl-2 antagonist of cell death) and antiapoptotic proteins (Bcl-2 family) decide the fate of the cell [13].

The current study is to investigate whether cardiac apoptosis in THS can be attenuated by early fluid resuscitation, and to understand the mechanism responsible for the effect. We hypothesize that THS may induce cardiac apoptosis via fas-dependent and mitochondrial dependent pathway; in addition, early fluid resuscitation may alleviate the cardiac apoptosis via IGF1/akt pathway.

## Materials and Methods

### Animals and groups

Male Sprague Dawley (SD) rats weighing 300~350 g were procured from National Science Council Animal Center, Taiwan. The rats were caged in ambient temperature of 25°C and on 12 h light-dark cycle. Rats were fed with standard laboratory chow (Lab Diet 5001; PMI Nutrition International Inc., Brentwood, MO, USA) and water. All protocols were approved by the Institutional Animal Care and Use Committee of China Medical University, Taichung, Taiwan, and the principles of laboratory animal care (NIH publication) were followed. The 21 rats were randomized into three groups: sham-operation group (Control, n = 3), hemorrhagic shock group (S, n = 9), hemorrhagic shock and resuscitation group (R, n = 9). In H and R groups, we killed 3 rats after resuscitation immediately (zero hour), four hours later and eight hours later respectively. The animals were continuously monitored throughout the procedure and vital signs such as the blood pressure and movement of chest were checked. The animals were euthanized by decapitation under terminal anesthesia and similar methods were in place for early euthanasia for animals that showed deterioration in the vital signs.

### Trauma-hemorrhagic shock

Male SD rats, weighing 300~350 g, were anesthetized using intraperitoneal injection by sodium pentobarbital (30 mg/kg). Following anesthesia, endotracheal intubation with 100% oxygen was supplied, we cannulated the right side femoral artery and the right side femoral vein with polyethylene catheters (PE50) filled with heparin. The mean arterial pressure (MAP) was recorded by a pressure transducer (Cardiomax-model 85; Columbus Instruments International Co., Ohio, U.S.A) which connected with the right femoral arterial catheter. Trauma was induced by mid-abdominal excision from xyphoid process to pubic bone, and re-sutured by layers with polyethylene.

### Hemorrhagic shock and resuscitation

In S and R groups, shock was induced by withdrawing blood (2.5 mL/kg) in 10 minutes to keep MAP 40±5 mm Hg. The shock duration was 60 minutes. In group H, the withdrawal blood was reinfused via the venous catheter in short time to maintain pre-operative MAP. In group R, administrated lactated Ringer’s solution (5 mL/kg) via the venous catheter in short time to maintain the MAP. After resuscitation, the arterial and venous vessels were sutured and PE50 were removed.

### Tissue Extraction

Left ventricle tissues were homogenized in PBS (0.14 M NaCl, 3 mM KCl, 1.4 mM KH_2_PO_4_, 14 mM K_2_HPO_4_) at a ratio of 100 mg tissue/0.5 mL. Proteins in the tissue homogenates were obtained as supernatant by centrifugation at 12,000 rpm for 30 min. The samples were collected and stored at -70°C until use.

### Terminal Deoxynucleotide Transferase-mediated dUTP Nick End Labeling (TUNEL)

The Left ventricle tissue sections were deparaffinized by xylene, and rehydrated. The sections were treated with proteinase K and rinsed in phosphate-buffered saline. The slides were then incubated in permeabilisation solution followed by incubation in blocking buffer, and then washed PBS twice. The tissue sections were then stained with TUNEL (Roche Applied Science, Indianapolis, IN, USA) for 60 min at 37°C. TUNEL-positive nuclei (fragmented DNA) were stained as bright green at 450–500 nm. The cell counts were performed by at least two independent individuals in a blinded manner.

### Electrophoresis and Western Blot

The protein concentration in left ventricular tissue extracts was determined using Bradford method (Bio-Rad Protein Assay, Hercules, CA). Protein samples (50 μg/lane) were separated in a 10% SDS polyacrylamide gel electrophoresis (SDS-PAGE) using 75 V. The proteins were then transferred to polyvinylidene difluoride (PVDF) membrane (Millipore, Bedford, MA, 0.45 μm pore size) and the PVDF membranes were blocked by incubating in 5% milk in TBS buffer. Primary antibodies Santa Cruz Biotechnology, Santa Cruz, CA, USA) were diluted to 1:500 overnight at 4°C. The blotted membranes were then washed thrice in TBS buffer for 10 min and immersed in appropriate secondary antibody solution (Santa Cruz) for 1 h and diluted 500-fold in TBS buffer. The membranes were then washed thrice in TBS buffer for 10 min. The proteins were visualized using a chemiluminescence ECL western Blotting luminal Reagent (Santa Cruz, CA, USA) and quantified using a Fujifilm LAS-3000 chemiluminescence detection system (Tokyo, Japan).

### Statistical Analysis

All the data were compared among the sham, hemorrhagic shock, and hemorrhagic shock with resuscitation groups using student T test. In all cases, *P*<0.05 was considered significant.

## Results

### Resuscitation with lactated Ringer’s solution shows early reduction of NFκB and TNF-α expression

**Fig 1:**
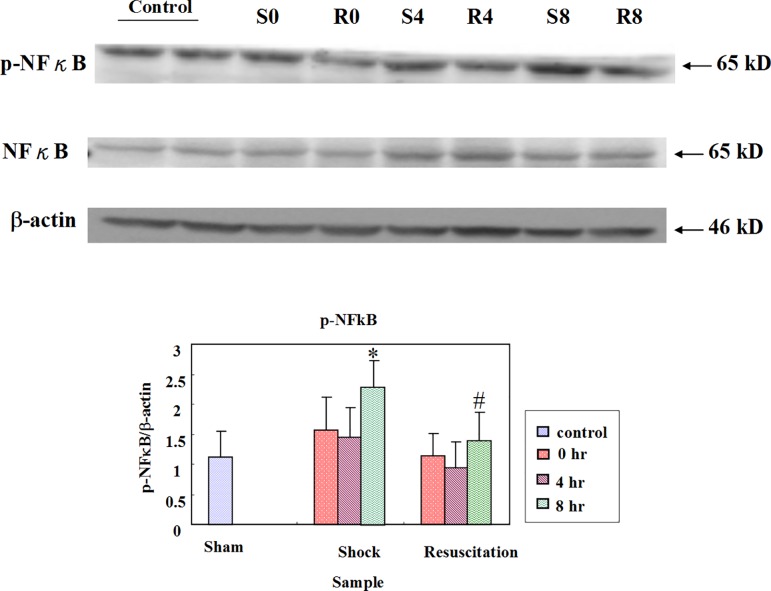
Effect of resuscitation with lactated Ringer’s solution on NFκB in rats with hemorrhagic shock. The protein levels of NFκB in the hearts of control rats, rats with hemorrhagic shock rats (S) and hemorrhagic shock rats resuscitated with lactated Ringer’s solution (R) were determined by western blotting analysis. β-actin was used as a loading control. * *P*< 0.05 denotes significant differences from control group and # *P*<0.05 denotes significant differences from hemorrhagic shock group

**Fig 2:**
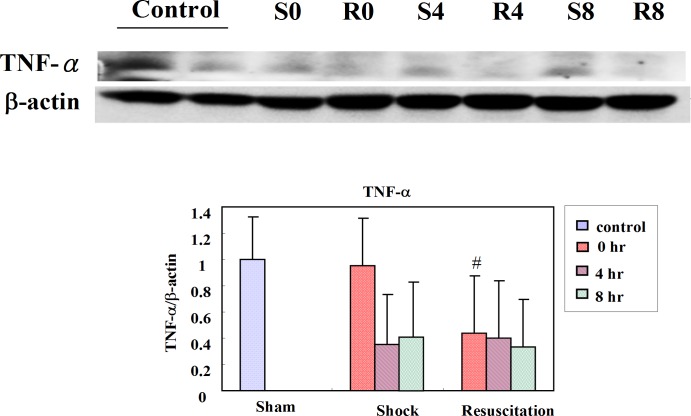
Effect of resuscitation with lactated Ringer’s solution on TNF-α in rats with hemorrhagic shock. The protein levels of TNF-α in the hearts of control rats, rats with hemorrhagic shock rats (S) and hemorrhagic shock rats resuscitated with lactated Ringer’s solution (R) were determined by western blotting analysis. β-actin was used as a loading control. # *P*<0.05 denotes significant differences from hemorrhagic shock group.

The NFκB and phosphorylated NFκB (p- NFκB) levels were measured after shock or shock with resuscitation at zero hour, four hours and eight hours. The p-NFκB/α actin level in R group was decreased in the control group and respectively at all time points in the S group. The NFκB levels at eight hours in the S group significantly increased with respect to the control group (p < 0.05), and the levels in the R group at eight hours decreased significantly when compared with the S group (p < 0.05) (Fig 1). The TNF-α level in the S group did not show any significant change compared to control group. The TNF-α level in the R group significantly decrease compared to H group at zero hour (Fig 2).

### Resuscitation with lactated Ringer’s solution suppresses Fas/FasL and FADD mediated apoptosis

**Fig 3:**
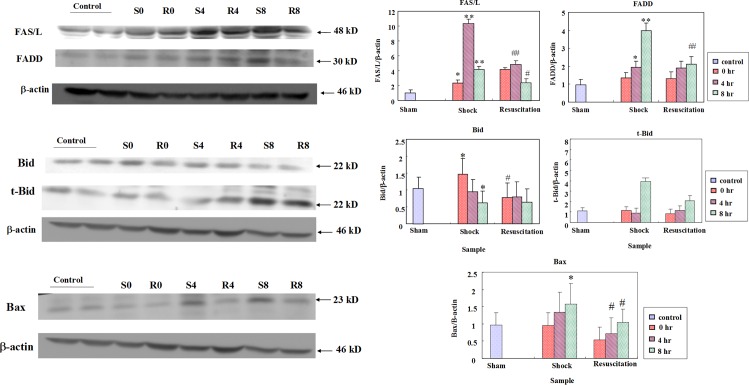
Effect of resuscitation with lactated Ringer’s solution on apoptosis associated proteins in rats with hemorrhagic shock. The protein levels of FAS/L, FADD, Bid, t-Bid, p-Bad, Bax in the hearts of control rats, rats with hemorrhagic shock rats (S) and hemorrhagic shock rats resuscitated with lactated Ringer’s solution (R) were determined by western blotting analysis. β-actin was used as a loading control. * and **—*P*< 0.05, *P*<0.01 denotes significant differences from control group and # # and ##- *P*< 0.05, *P*<0.01 denotes significant differences from hemorrhagic shock group

The FasL levels in the S group elevated significantly after hemorrhagic shock when compared to the control group, especially at 4 h (p <0.01). In addition to FasL, the level of FADD also was significantly enhanced in the S group than in the control group at 4 h (p < 0.05) and 8 hours (p< 0.01). However, after resuscitation the FasL and FADD levels were significant decreased as seen in the R group (Fig 3). Hemorrhagic shock also modulated the levels of other apoptosis associated proteins such as tBid, Bax, pBad, Bax in the S group at all the time points (Fig 3). But the levels were regulated in the R groups which received lactated Ringer’s solution.

### Resuscitation with lactated Ringer’s solution ameliorates cytochrome *c* release triggered by Hemorrhagic shock

**Fig 4:**
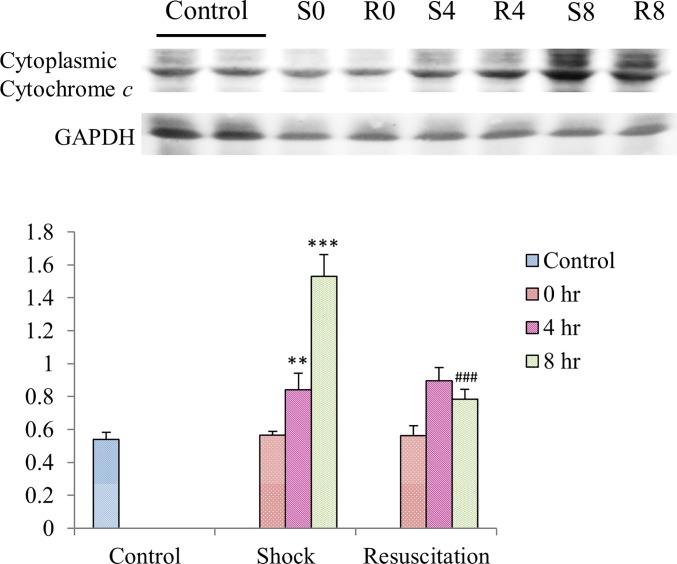
Effect of resuscitation with lactated Ringer’s solution on cytochrome *c* release in rats with hemorrhagic shock. The protein levels of cytochrome *c* in the cytoplasm of cardiomyocytes of control rats, rats with hemorrhagic shock rats (S) and hemorrhagic shock rats resuscitated with lactated Ringer’s solution (R) were determined by western blotting analysis. GAPDH was used as a control. ** and ***—*P*< 0.01, *P*<0.001 denotes significant differences from control group and ### *P*< 0.001 denotes significant differences from hemorrhagic shock group.

Western blot analysis of the cytoplasmic proteins show an increased level of cytochrome *c* in the cytoplasm of cardiomyocytes of S group rat hearts whereas the lactated Ringer’s solution reduced the mitochondrial damage as seen from the reduced cytochrome *c* release in the R group (Fig 4).

### Resuscitation with lactated Ringer’s solution attenuates apoptosis induced by Hemorrhagic shock

**Fig 5:**
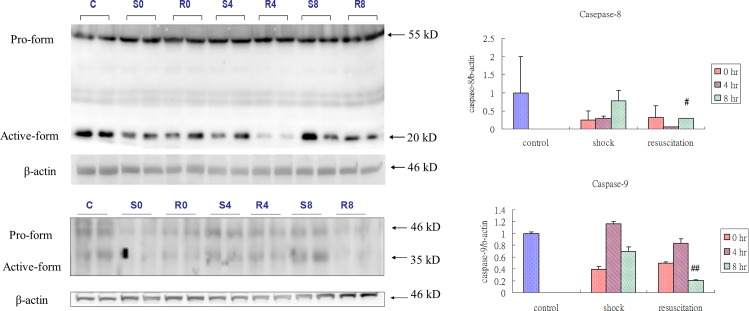
Effect of resuscitation with lactated Ringer’s solution on active caspases in rats with hemorrhagic shock. The protein levels of caspase-8 and caspase-9 in the hearts of control rats, rats with hemorrhagic shock rats (S) and hemorrhagic shock rats resuscitated with lactated Ringer’s solution (R) were determined by western blotting analysis. β-actin was used as a loading control. * *P*< 0.05 denotes significant differences from control group and ## *P*<0.01 denotes significant differences from hemorrhagic shock group.

**Fig 6:**
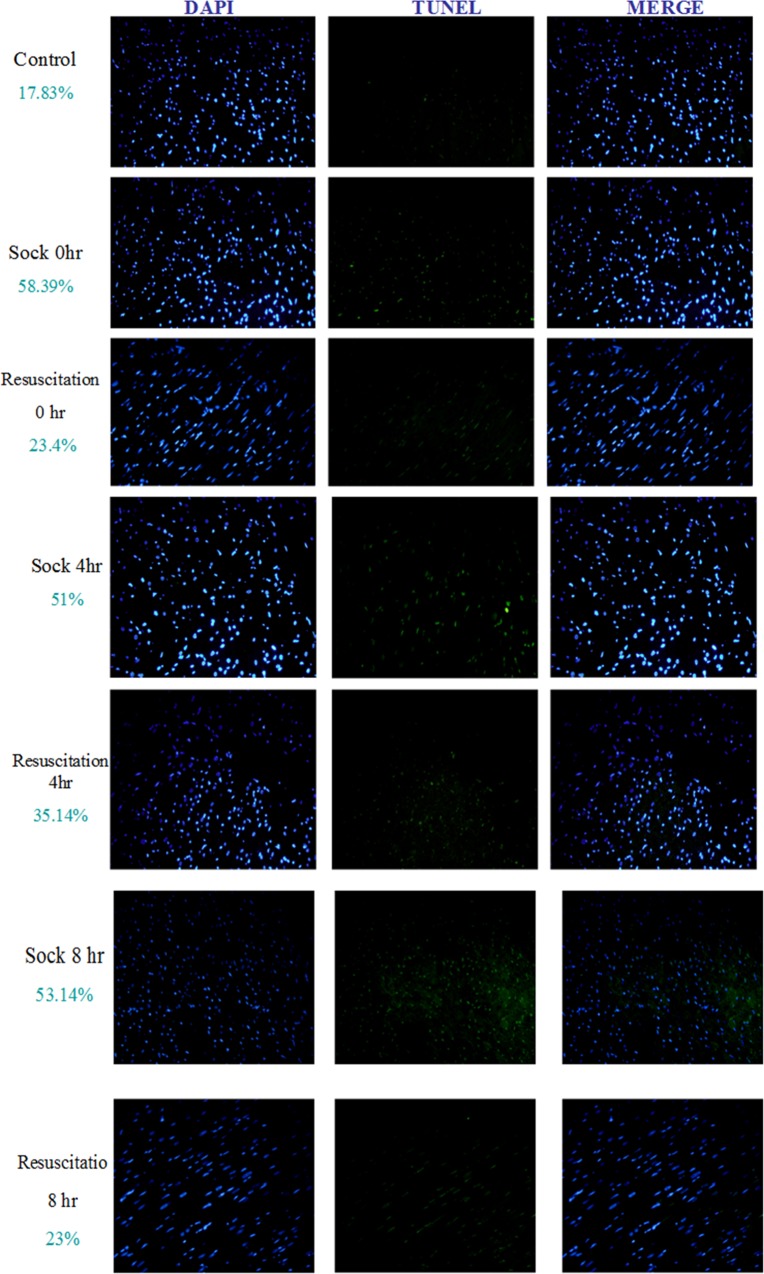
Effect of resuscitation with lactated Ringer’s solution on cardiomyocyte apoptosis in rats with hemorrhagic shock. DAPI and TUNEL stained heart sections of control rats, rats with hemorrhagic shock rats (S) and hemorrhagic shock rats resuscitated with lactated Ringer’s solution (R). The nuclei were stained in blue color after DAPI staining, and DNA fragments caused by apoptosis process were stained in green color after TUNEL assay.

Further the levels of cleaved caspase-8 and cleaved caspase-9 were also suppressed significantly 8 h after Resuscitation with lactated Ringer’s solution (Fig 5). TUNEL staining revealed that the number of TUNEL positive nuclei increased after hemorrhagic shock in the S group rat hearts. In contrast, lactated Ringer’s solution effectively alleviated the apoptotic response (Fig 6).

### Resuscitation with lactated Ringer’s solution enhances IGF-IR/AKT associated cell survival pathway

**Fig 7:**
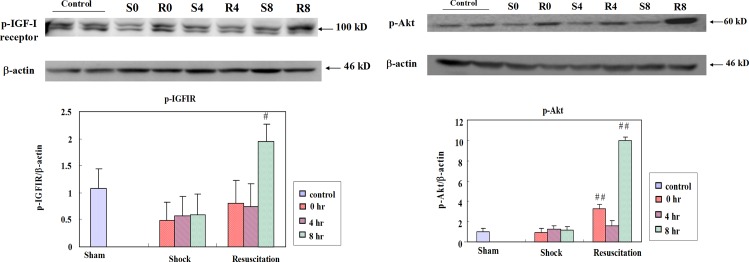
Effect of resuscitation with lactated Ringer’s solution on IGF-IR survival mechanism in rats with hemorrhagic shock. The protein levels of IGF-IR, pAkt in the hearts of control rats, rats with hemorrhagic shock rats (S) and hemorrhagic shock rats resuscitated with lactated Ringer’s solution (R) were determined by western blotting analysis. β-actin was used as a loading control. ## *P*<0.01 denotes significant differences from hemorrhagic shock group.

The levels of pIGF-IR and pAkt that were suppressed after hemorrhagic shock in the S group were significantly enhanced after resuscitation with lactated Ringer’s solution particularly, 8 h after the treatment (Fig 7).

## Discussion

Trauma-hemorrhagic shock leads to an insufficiency between tissue demand and supply causing hypoxia and metabolic acidosis. Attending and treating hemorrhagic shock immediately after traumatic injury has become much critical for modern trauma care [14]. Current guidelines for presurgical treatment recommend rapid administration of volume resuscitation to bring back normal blood pressure readily. Controlled fluid resuscitation enables treatments during prehospital care to work in a compensatory mechanism. The strategy involves restoration of some intravascular fluid to attain a point of deliberate hypotension [15]. The major causes of death during late stage of patients with severe hemorrhagic shock are infection, multiple organ dysfunction syndromes and/or multiple organ failure. Researchers now recognize that apoptosis is generally triggered in visceral organs during the early stage of hemorrhagic shock, and is a major reason for initial organ damage and subsequent multiple organ failure [16].

In this study, we recorded the pathological changes in myocardial cells during hemorrhagic shock, and examined the mechanisms by which resuscitation with lactated Ringer’s solution exerts protective effects in a rat model.

In a randomized controlled pilot trial points out that early resuscitation with the large amounts of pH-balanced lactated Ringer's solution reduces prevalence of systemic inflammatory response syndrome as seen in patients with acute pancreatitis during the first 24 h of hospitalization in comparison with resuscitation with normal saline [17].

In a separate study on an uncontrolled hemorrhagic shock animal model, the plasma chloride concentration was found to be significantly lower in resuscitation with lactated Ringer’s solution. Further the resuscitation with lactated Ringer’s solution causes significant lactatemia which is independent of acidosis therefore lactated Ringer's solution has been hypothesized to be superior to normal saline for the resuscitation of uncontrolled hemorrhagic shock [18].

Our results clearly show that resuscitation with lactated Ringer’s solution reduces the number of TUNEL positive nucleus in the hearts of rats under hemorrhagic shock, which suggests that lactated Ringer’s solution may exert the protective roles through apoptosis signaling.

It is well-known phenomenon that apoptosis is triggered by two basic signaling pathways-the extrinsic and the intrinsic pathways. The extrinsic pathway involves activation by death ligands, such as the Fas and FasL, binding of which results in recruitment of adapter proteins and initiation of caspases. In this study, we observed a notable increase in levels of Fas and FasL, and in the levels of caspase-8 and caspase-9 expression [19]. Resuscitation with lactated Ringer’s solution remarkably attenuated the increasing trend of these molecular markers. The results suggest that the myocardiocyte apoptosis induced by hemorrhagic shock may is mediated via extrinsic pathways, and resuscitation with lactated Ringer’s solution attenuates the apoptosis by interrupting the Fas/FasL/caspase-8 signal pathway.

This is one of the few studies to investigate the cardio-protective roles of resuscitation with lactated Ringer’s solution in hemorrhagic shock, and is also the first research to reveal that lactated Ringer’s exerts the effect via the possible extrinsic Fas/FasL/caspase-8 involved apoptosis signal pathways.

IGF-I ligand is also known to improve cardiomyocyte function and rescues from heart failure [20]. The IGF-I mediated signaling pathway mediates the inhibition of apoptosis in the myocardium and in various other cells. Our results also show that lactated Ringer’s solution enhances the survival mechanism in the rat heart.

## Conclusion

Our *in vivo* study showed that resuscitation with lactated Ringer’s solution ameliorates hemorrhagic shock triggered hemodynamic deterioration by decreasing myocardial apoptosis. The results suggested that lactated Ringer’s might exert its cardio-protective roles via attenuating the extrinsic Fas/FasL/caspase-8 involved apoptosis pathways and by enhancing IGF-IR/Akt cell survival pathway.
