# Correction: Early Fluid Resuscitation by Lactated Ringer's Solution Alleviate the Cardiac Apoptosis in Rats with Trauma-Hemorrhagic Shock

**DOI:** 10.1371/journal.pone.0168419

**Published:** 2016-12-09

**Authors:** Kuan-Ho Lin, Chien-Liang Liu, Wei-Wen Kuo, Catherine Reena Paul, Wei-Kung Chen, Su-Ying Wen, Cecilia Hsuan Day, Hsi-Chin Wu, Vijaya Padma Viswanadha, Chih-Yang Huang

The affiliation for the second author is incorrect. Chien-Liang Liu is not affiliated with #3 but with #2 Department of Emergency Medicine, China Medical University Hospital, Taichung, Taiwan.
